# Efficacy and safety of obinutuzumab in refractory membranous nephropathy: a single-arm meta-analysis

**DOI:** 10.3389/fimmu.2026.1880420

**Published:** 2026-07-21

**Authors:** Pengli Cong, Jiayang Wang, Xuanyi Du

**Affiliations:** Department of Nephrology, The Second Affiliated Hospital of Harbin Medical University, Harbin, Heilongjiang, China

**Keywords:** biologics, efficacy, obinutuzumab, refractory membranous nephropathy, safety

## Abstract

**Objective:**

Membranous nephropathy (MN) constitutes an autoimmune glomerular disorder and represents the most frequent etiology of primary nephrotic syndrome in adults. Although obinutuzumab exhibits favorable efficacy in managing refractory MN, outcomes diverge across individual investigations. Consequently, the present meta-analysis is undertaken to systematically evaluate the clinical efficacy and safety of obinutuzumab in individuals with refractory MN.

**Methods:**

A systematic search was conducted across the Cochrane Library, Embase, Web of Science, and PubMed to determine relevant clinical investigations published up to November 12, 2025. Treatment effects were quantified employing the pooled proportion rate with 95% confidence interval (CI), mean difference (MD, 95% CI), and standardized mean difference (SMD, 95% CI). Heterogeneity was examined via the I² statistic. Subgroup analyses were implemented based on the geographic distribution of the study population and duration of follow-up. All extracted data were subjected to statistical analysis using R (v4.5.2).

**Results:**

The present meta-analysis ultimately incorporated 12 articles (222 individuals) after applying the eligibility criteria. Concerning remission rates, the overall clinical remission rate reached 86% (95% CI: 80% to 90%), the complete clinical remission rate stood at 31% (25% to 37%), the partial clinical remission rate was 55% (49% to 63%), and the immunologic remission rate achieved 88% (81% to 92%). Obinutuzumab reduced proteinuria (SMD = −1.2, 95%CI −1.37 to −1.03) and raised serum albumin (MD = 12.5, 95%CI 9.91–15.08), while eGFR showed no significant change (MD = 3.23, 95%CI −3.76–10.21). The pooled adverse event rate was 29% (95%CI 15%–48%), with two fatal pneumonia-related events: severe COVID-19 pneumonia and pneumonia-induced respiratory failure.

**Conclusion:**

Obinutuzumab demonstrates marked efficacy in treating refractory MN, with an overall clinical remission rate of 86% and a low incidence of severe adverse events. This profile signifies a potential therapeutic role for this condition. However, all raw data included in this meta-analysis were derived from uncontrolled retrospective single-arm trials and case series. Given factors such as selection bias, the remission rate may be overestimated. Large-scale, multicenter randomized controlled trials are necessary for verification in future investigations.

**Systematic review registration:**

https://www.crd.york.ac.uk/PROSPERO/, identifier CRD420251231344.

## 1 Introduction

Membranous nephropathy (MN) is an autoimmune glomerular disease pathologically marked by thickening of the glomerular capillary wall resulting from the deposition of an immune complex. MN may manifest at any age and constitutes the most common cause of primary nephrotic syndrome in adult individuals. No discernible etiology is identified in approximately 80% of affected individuals, a condition designated primary membranous nephropathy (PMN). In the remaining cases, the pathogenesis is linked to agents or concomitant disorders—such as systemic lupus erythematosus, hepatitis viral infection, or malignancy—classified as secondary MN (1). PMN represents the foremost cause of non-diabetic adult idiopathic nephrotic syndrome worldwide, with an incidence reaching 58% among adult individuals aged above 65 years. The clinical trajectory of PMN exhibits considerable variability. Roughly one-third of untreated individuals attain spontaneous remission. Another third may progress to end-stage renal disease within a decade. The remainder typically enter a non-progressive phase of chronic kidney disease (1–3). Currently, large-scale population studies representative of diverse international cohorts are still lacking. Thus, data regarding the incidence of MN and its subtypes remain notably constrained. The annual incidence of MN is estimated at 10% to 12% in North America and 2% to 17% in Europe (1). Among glomerular diseases in China, the incidence of MN stands at 23.4%, ranking second and exhibiting a trend toward surpassing that of immunoglobulin A nephropathy (4).

Over the preceding two decades, substantial advancements have been achieved in elucidating the pathophysiology of MN. In 2009, the M-type phospholipase A2 receptor (PLA2R) was identified as a new podocyte antigen, serving as the principal antigenic target implicated in the pathogenesis of adult PMN. Anti-PLA2R antibodies represent a specific serologic marker for MN, detectable in around 70% of adult cases (5). Subsequent discovery of additional target antigens, including thrombospondin type-1 domain-containing 7A, has further refined comprehension of the pathogenesis of PMN (6) and facilitated the broader application of immunosuppressors. Conventional immunosuppressive therapies exhibit limited efficacy, and their associated adverse effects and toxicity profiles restrict long-term administration (7, 8). As knowledge concerning the role of B lymphocytes in the pathogenesis of MN has expanded, rituximab (RTX)—a chimeric murine/human monoclonal antibody specifically targeting the CD20 antigen expressed on B cells—has become a first-line therapeutic intervention. Nevertheless, 20% to 40% of individuals fail to respond to an initial course of RTX (9). A pressing clinical need, therefore, exists for novel therapeutic strategies directed toward refractory MN.

Obinutuzumab is a humanized type II anti-CD20 monoclonal antibody. Relative to type I anti-CD20 monoclonal antibodies such as RTX, obinutuzumab exhibits enhanced antibody-dependent cellular cytotoxicity, augmented capacity for direct B-cell killing, and reduced reliance on complement-dependent cytotoxicity (10). Multiple investigations have documented the favorable efficacy of obinutuzumab in managing refractory MN. Nonetheless, reported outcomes display inter-study variability. The investigation by Dong et al. (11)reports an overall clinical remission rate (OR rate) of 100%, whereas the study by Li, L et al. (12)observes a rate of 76%. Concurrently, the incidence of adverse events (AEs) attributed to obinutuzumab also demonstrates pronounced divergence across studies (13, 14). Comprehensive evidence derived from systematic reviews and single-arm meta-analyses remains lacking. Thus, the present meta-analysis is designed to integrate existing clinical data and systematically examine the clinical efficacy—including OR rate, complete clinical remission rate (CR rate), partial clinical remission rate (PR rate), and immunologic remission rate (IR rate), and alterations in proteinuria—and safety profile—encompassing incidences of infection, infusion-related reactions, and other AEs—of obinutuzumab in individuals with refractory MN. The aim is to furnish evidence-based guidance for clinical decision-making, establish a foundation for future large-scale randomized controlled trials (RCTs), and ultimately enhance therapeutic outcomes and quality of life for affected individuals.

## 2 Methods

The current meta-analysis adhered to the Preferred Reporting Items for Systematic Reviews and Meta-Analyses guidelines (15). The protocol was registered with the International Prospective Register of Systematic Reviews (CRD420251231344).

### 2.1 Literature search

Four electronic databases—the Cochrane Library, Web of Science, Embase, andPubMed—were systematically searched up to November 12, 2025. Relevant subject headings and free-text terms were determined via a MeSH query. Search strategies were constructed employing Boolean operators (AND, OR) with the following keywords: obinutuzumab, membranous nephropathy. **Supplementary File 1** provides the details. Additionally, reference lists of eligible articles were manually screened.

### 2.2 Eligibility criteria

The Evidence-Based Medicine PICOS framework (participants, interventions, comparators, outcomes, study design) was applied to formulate our eligibility criteria.

Inclusion criteria comprised: (i) Participants: individuals clinically diagnosed with refractory MN. The current study defined refractory MN as follows: persistently high or unchanged levels of PLA2R antibodies after adequate doses and courses of first-line immunosuppressive therapy, or nephrotic syndrome with persistently low serum albumin levels lasting more than 6 months during follow-up. (ii) Intervention: obinutuzumab; (iii) Outcomes: reporting of at least one of the following—OR, CR, PR, and IR rates (supplementary definitions provided in Appendix 4), or incidence of AEs; (iv) Study design: single-arm clinical trial, cohort studies, or case series.

Exclusion criteria encompassed: (i) publications classified as meta-analyses, animal experiments, conference abstracts, reviews, or commentaries; (ii) duplicate publications; (iii) articles with unavailable full texts or incomplete data.

### 2.3 Literature screening and data extraction

CPL and WJY separately screened the retrieved records within EndNote 2025 per predefined eligibility criteria. Initial screening involved evaluation of titles and abstracts to determine possibly pertinent articles, followed by retrieval of full texts. A subsequent full-text review was conducted to ascertain articles for final inclusion and data extraction. Collected information comprised publication year, first author, sample size, country, proportion of male sex, intervention, OR, CR, PR, and IR rates, and laboratory parameters. For continuous variables—24-hour urine protein (24hUP) or urinary protein-to-creatinine ratio (UPCR), serum albumin (SA), serum creatinine (SCr), and estimated glomerular filtration rate (eGFR)—the mean and standard deviation (SD) at baseline (pre-treatment) and at the follow-up endpoint (post-treatment) were extracted from each study. The mean change for each variable was calculated directly by subtracting the baseline mean from the endpoint mean. When the SD of the change was not reported in the original article, it was estimated according to the methods recommended in the Cochrane Handbook for Systematic Reviews of Interventions (Higgins JPT, Thomas J, Chandler J, Cumpston M, Li T, Page MJ, et al. Cochrane Handbook for Systematic Reviews of Interventions version 6.5 (updated August 2024). Cochrane, 2024). For continuous variables measured in uniform units (SA, SCr, eGFR), the mean difference (MD) was adopted as the pooled effect measure. Because the methods and units for quantifying proteinuria were inconsistent (g/24 h versus g/g), the standardized mean difference (SMD) was chosen for pooled analysis to eliminate unit heterogeneity. When the aforementioned data were not directly available, they were calculated based on information provided within the articles. Discrepancies were resolved through consultation with DXY.

### 2.4 Quality evaluation

CPL and WJY independently appraised the methodological quality of eligible articles utilizing the methodological index for non-randomized studies (MINORS) instrument. MINORS comprises 12 items. The initial 8 items apply to single-arm investigations, whereas all 12 items pertain to comparative studies. Each item receives a score of 0–2. Domains evaluated include a clearly stated aim, consecutive inclusion of participants, prospective collection of data, appropriateness of endpoints relative to the study aim, unbiased assessment of endpoints, adequacy of follow-up duration, loss to follow-up below 5%, and consideration of sample size estimation. For single-arm studies, scores below 8, of 9–12, and of 13–16 denoted low, moderate, and high quality, respectively. For comparative studies, scores below 12, of 13–18, and of 19–24 denoted low, moderate, and high quality, respectively. Discrepancies were resolved through discussion with DXY.

### 2.5 Statistical analysis

Therapeutic efficacy was analyzed employing pooled proportion rates with corresponding 95% confidence intervals (CIs). Statistical analyses for all collected data (e.g., remission rates, adverse event incidence rates, MD, and SMD) were implemented in R (v4.5.2). MD denotes the absolute change in continuous variables from baseline to post-treatment measurement and is applicable when the units of measurement remain uniform across investigations. MD directly reflects the mean magnitude of alteration. SMD represents the MD divided by the corresponding SD, thereby eliminating discrepancies in unit or scale. SMD is adopted when the same variable is measured in different units or via disparate evaluation tools across investigations. In the present meta-analysis, MD was employed for pooling pre- and post-treatment changes in continuous variables with uniform units (SA, eGFR, SCr). As the contributing studies reported proteinuria using different methods and units—some as 24-hour urine protein (g/24h), others as UPCR (g/g)—no reliable conversion formula applicable at the aggregate data level (mean, SD) currently exists for individuals with proteinuria exceeding 3 g/d. This meta-analysis therefore employed the SMD as the pooled effect measure for change in proteinuria. For each included investigation, SMD was calculated by dividing the mean change by the SD of the change. This removed the influence of disparate measurement units and enabled results from studies with different units to be synthesized on a common scale. The magnitude of the pooled effect was interpreted using Cohen’s criteria: negligible or very small effect for |SMD|< 0.2, small effect for 0.2 ≤ |SMD|< 0.5, medium effect for 0.5 ≤ |SMD|< 0.8, and large effect for |SMD|≥ 0.8 (Higgins JPT, Thomas J, Chandler J, Cumpston M, Li T, Page MJ, et al. Cochrane Handbook for Systematic Reviews of Interventions version 6.5 [updated August 2024]. Cochrane, 2024). Inter-study heterogeneity was quantified via the I² statistic. I² exceeding 50%, coupled with p < 0.1, indicated substantial heterogeneity, and a random-effects model was adopted; otherwise, a fixed-effects model was utilized. Subgroup analyses were implemented based on follow-up duration and geographic region. Publication bias was evaluated via funnel plots and Egger’s test. Sensitivity analyses were implemented to ascertain the stability and reliability of the findings. Statistical significance was set at p < 0.05.

## 3 Results

### 3.1 Literature screening

**Figure 1:**
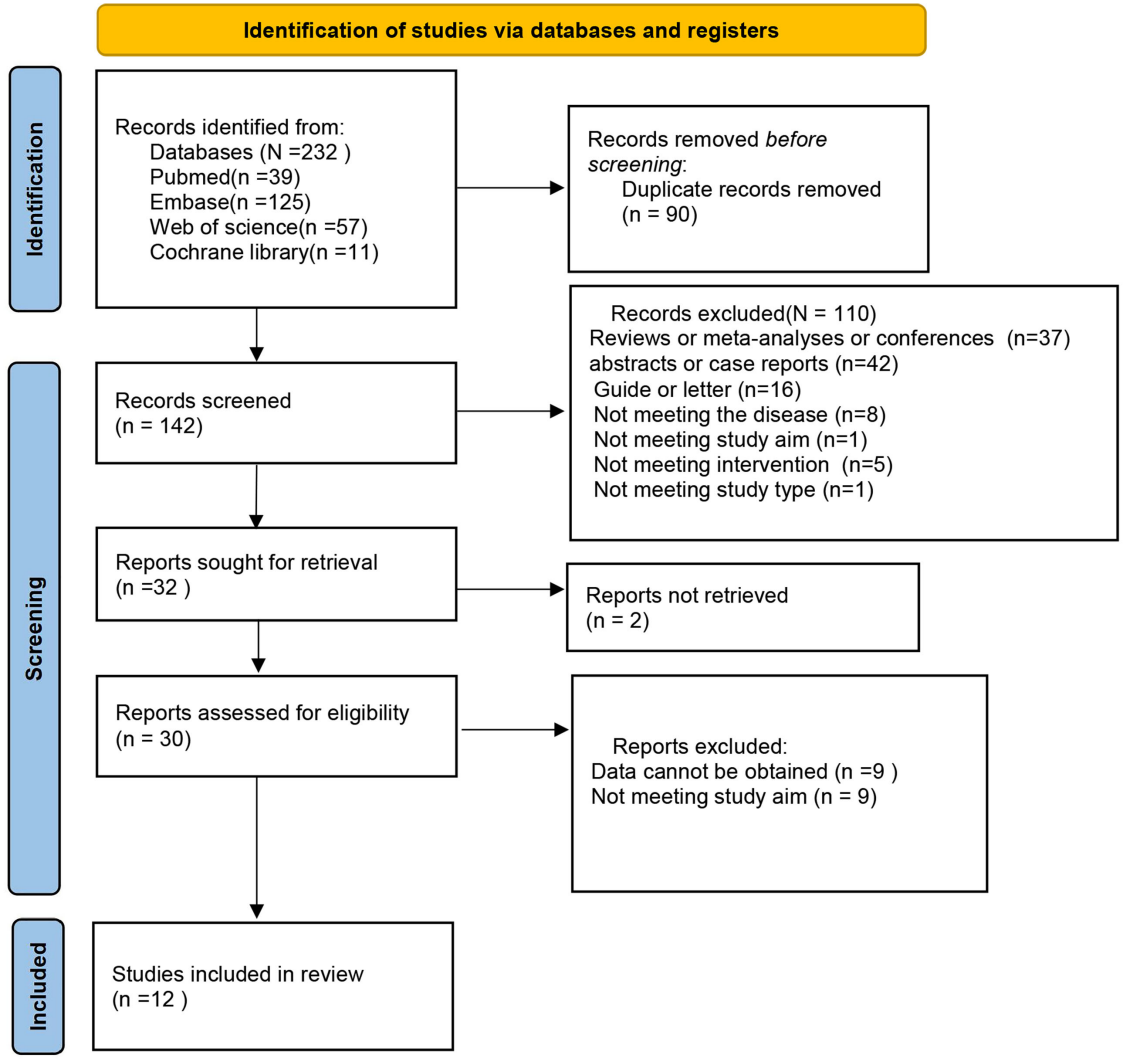
Study flow diagram.

The initial search yielded 232 relevant records. After eliminating 90 duplicates, 110 records were removed by screening titles and abstracts. Full text could not be obtained for 2 records, and 18 records were eliminated upon full-text evaluation. Ultimately, 12 studies were incorporated (10–14, 16–22). The screening process is depicted in **Figure 1**.

### 3.2 Characteristics of literature

Included publications spanned the period from 2020 to 2025. Nine studies originated from China. The remaining three were conducted in the United States (10), Australia (14), and India (21), respectively. Two investigations employed a cohort design (17, 19); all others were single-arm studies. Encompassing a total of 222 individuals, the study population had a mean age ranging from 38.5 to 58.0 years, a male proportion that varied between 60% and 94.9%, and a mean disease duration extending from 9 to 85 months. Prior therapeutic regimens comprised glucocorticoids, RTX, cyclophosphamide, calcineurin inhibitors, and mycophenolate mofetil, among others. Baseline characteristics of eligible publications are summarized in **Table 1**.

**Table 1:** Characteristics of included studies.

Id	Author	Year	Country	Design	Sample size	Age	Male/female	Type of disease	Disease duration (months)	Prior treatment	Follow-up duration (months)	Obinutuzumab dosing regimen
							Proportion of male sex					
1	Su	2025	China	Single-arm clinical trail	20	46.25 ± 13.05	70%	Rituximab-refractory PLA2R-associated primary membranous nephropathy	9.26± 4.18	RTX:100%; CNIs:55%; Glucocorticoid: 60%; CTX: 25%; Mycophenolate mofetil:5%	5.31± 4.18	Initial: two 1 g obinutuzumab infusions, 2 weeks apart. At month 6: supplemental 1–2 g obinutuzumab for 5 patients with urine protein >3.5 g/day and 4 patients with 6-month B-cell reconstitution.
2	Xu	2025	China	Retrospective cohort study	20	52.58± 12.05	75%	Refractory primary membranous nephropathy (95% PLA2R-associated)	32.61 ± 30.48	corticosteroid+CTX:150%; CNIs+corticosteroid: 65%	22.56 ± 19.15	Obinutuzumab administered intravenously at a dose of 1 g, given in 1–2 administrations with at least a 2-week interval between doses.
3	Li, L	2025	China	Single-arm clinical trail	25	44.00 ± 12.93	84%	Refractory primary membranous nephropathy (84% anti-phospholipase A2 receptor antibody positive)	26.50 ± 22.79	RTX: 84%; corticosteroid+CTX:36%; corticosteroid+CNIs:40%; CNIs:60%; mycophenolate mofetil:4%	12.72 ± 1.57	All patients received obinutuzumab with single dose of 1000 mg, total cumulative doses: 1000 mg (3 patients), 2000 mg (12 patients), 3000 mg (8 patients), 4000 mg (2 patients).
4	Li, H	2025	China	Single-arm clinical trail	17	49.70 ± 13.70	64.70%	Rituximab-refractory PLA2R-associated primary membranous nephropathy	22.84 ± 24.25	RTX:100%; RTX+other immunosuppressants: 41.2%	12.6 ± 5.00	Two 1 g obinutuzumab infusions,2 weeks apart. Additional obinutuzumab infusions were administered during follow-up based on changes in clinical parameters.
5	Cheng	2025	China	Retrospective cohort study	40	55.20 ± 13.30	70%	Refractory primary membranous nephropathy	NA	RTX:75%; CNIs:57.5%; CTX:22.5%; other: 25%	13.99 ± 6.77	Two 1 g obinutuzumab infusions, 2 weeks apart.
6	Chen	2025	China	Single-arm clinical trail	7	57.01± 26.64	71%	Rituximab-refractory PLA2R-associated primary membranous nephropathy	24.13 ± 21.13	RTX:100%; CNIs:86%; CTX:57%	11.78 ± 12.86	Two 1 g obinutuzumab infusions, at least 2 weeks apart. Infusion frequency was determined jointly by physician and patient following professional consultation.
7	Sethi	2020	U.S.	Single-arm clinical trail	10	57.90± 16.33	40%	Refractory membranous nephropathy	NA	RTX:70%; CNI:50%; CTX: 20%; mycophenolate mofetil: 40%; corticosteroid:70%	16.89 ± 12.90	Not reported.
8	Dong	2025	China	Single-arm clinical trail	15	58.00 ± 13.09	66.70%	Refractory primary membranous nephropathy ( 93.3% PLA2R-associated)	85.49 ± 113.69	RTX:73.3%; CNIs+corticosteroid:13.3% CTX+corticosteroid:46.7%	9.34 ± 2.29	Two 1 g obinutuzumab infusions, 2 weeks apart. Administration frequency. Individualized by clinicians following KDIGO guidelines.
9	Su	2024	China	Single-arm clinical trail	39	51.90 ± 14.00	94.90%	Refractory primary membranous nephropathy (84.6% PLA2R-associated)	NA	RTX:35.9%; CNI:71.8%; corticosteroid:84.6%; CTX:28.2%; Tripterygium wilfordii: 7.7%; mycophenolate mofetil: 2.6%	9.50 ± 2.69	Two 1 g obinutuzumab infusions, 2–3 weeks apart.
10	Jha	2025	India	Single-arm clinical trail	6	38.50 ± 9.44	83.30%	Refractory PLA2R-associated primary membranous nephropathy	NA	RTX:100%; CNI:100%, CTX+corticosteroid:50%	12.00 ± 3.16	Two 1 g obinutuzumab infusions, 2 weeks apart.
11	Lin	2024	China	Single-arm clinical trail	18	50.48 ± 7.96	83.30%	Refractory primary membranous nephropathy (83.3% PLA2R-associated)	12.86 ± 14.72	RTX:66.7%; CTX:44.4%; corticosteroid:77.8%; mycophenolate mofetil: 33.3%; CNI:33.3%	13.46 ± 2.41	Two 1 g obinutuzumab infusions,2 weeks apart. Initial cycle ends when CD19+ B cells reach BCD (0 cells/μL); if not, extra 1 g infusions every 2 weeks until BCD is achieved.
12	Sridharan	2025	Australia	Single-arm clinical trail	5	53.60 ± 17.87	60%	Refractory membranous nephropathy (80% PLA2R-associated)	NA	RTX:100%; CNI:80%; corticosteroid:60%; mycophenolate mofetil: 20%; azathioprine: 20%	9.91 ± 4.23	Standard obinutuzumab regimen: 100 mg test dose (Day 1), 900 mg (Day 2), 1000 mg (Day 15).

RTX, Rituximab; CNI, Calcineurin inhibitor; CNIs, Calcineurin inhibitors; CTX, Cyclophosphamide.

Values for Age, Disease duration, and Follow-up duration are expressed as mean ± standard deviation(SD).

NA, not available (data were not reported or could not be extracted).

### 3.3 Quality evaluation

Methodological quality of the 12 eligible articles was evaluated employing the MINORS instrument. Two investigations were rated as high quality (17, 19), and ten as moderate quality. All eligible investigations were retrospective, did not entail prospective collection of data, and omitted estimation of the requisite sample size. Consecutive enrollment of patients was suboptimal in three studies (10, 14, 21). Follow-up duration was deemed inadequate in three publications (14, 18, 20). Loss to follow-up exceeded 5% in one study (10). The comparator intervention was considered inappropriate in two cohort studies (17, 19). Details are presented in **Table 2**.

**Table 2:** Assessment of research quality.

Study	A clearly stated aim	Inclusion of consecutive patients	Prospective collection of data	Endpoints appropriate to the aim of the study	Unbiased assessment of the study endpoint	Follow-up period appropriate to the aim of the study	Loss to follow-up <5%	Prospective calculation of the study size	An adequate control group	Contemporary groups	Baseline equivalence of groups	Adequate statistical analyses	Total
(16)	2	2	0	2	2	2	2	0					12
(17)	2	2	0	2	2	2	2	0	1	2	2	2	19
(12)	2	2	0	2	2	2	2	0					12
(12)	2	2	0	2	2	1	2	0					11
(19)	2	2	0	2	2	2	2	0	1	2	2	2	19
(20)	2	2	0	2	2	1	2	0					11
(10)	2	1	0	2	2	2	0	0					9
(11)	2	2	0	2	2	2	2	0					12
(13)	2	2	0	2	2	2	2	0					12
(21)	2	1	0	2	2	0	2	0					9
(22)	2	2	0	2	2	2	2	0					12
(14)	2	1	0	2	2	1	2	0					10

### 3.4 Meta-analysis

#### 3.4.1 Remission rates

##### 3.4.1.1 OR rate

**Figure 2:**
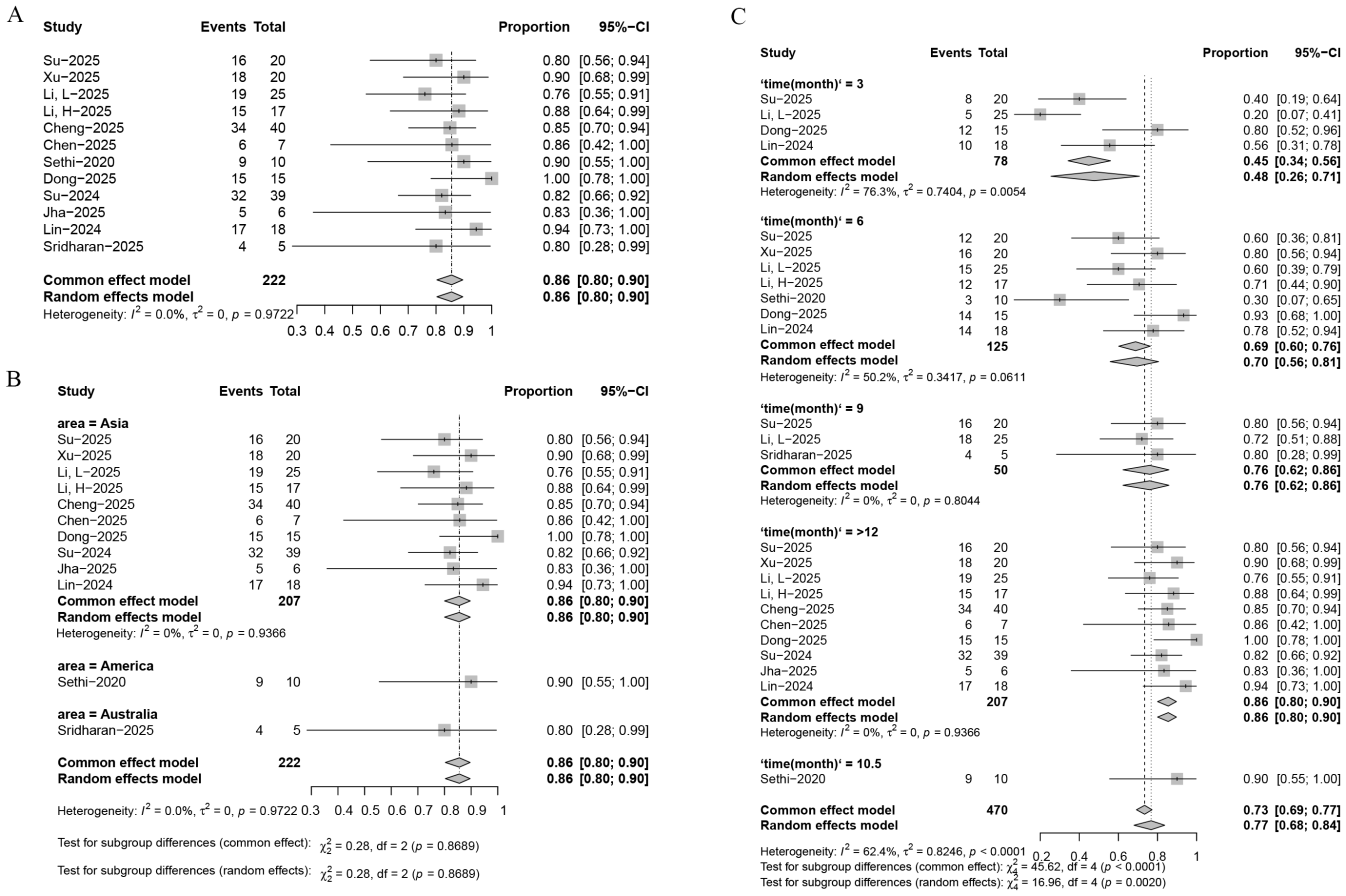
**(A)** Forest plot of the overall clinical remission rate; **(B)** Forest plot of the subgroup analysis of area for the overall clinical remission rate; **(C)** Forest plot of the subgroup analysis of follow-up time for the overall clinical remission rate.

Twelve investigations evaluated the OR rate. Inter-study heterogeneity was low (I² = 0.0%, p = 0.972), and a fixed-effects model was therefore employed. The pooled OR rate reached 86% (95% CI: 80% to 90%; **Figure 2A**). Subgroup analyses based on geographic region and follow-up duration were implemented. Following stratification by region, the OR rate was 86% (80% to 90%) in Asia. For single studies from America and Australia, only the individual study results were presented: 90% (10) and 80% (14), respectively (**Figure 2B**). Because only one study was available from each of these regions, the samples lacked representativeness, and the findings could not be generalized to the broader American or Australian populations. After stratification by follow-up duration, the pooled OR rate was 48% (26% to 71%) at 3 months, 70% (56% to 81%) at 6 months, 76% (62% to 86%) at 9 months, 90% (55% to 100%) at 10.5 months, and 86% (80% to 90%) at ≥ 12 months (**Figure 2C**). Regression analyses assessing associations of OR rate with sex and pre-treatment laboratory parameters (24hUP, SA, SCr, eGFR) revealed no marked correlations (**Table 3**). Given the modest number of contributing studies, statistical power was limited. The absence of significant associations observed in these regression analyses should therefore be regarded as exploratory and lacking confirmatory inferential value.

**Table 3:** Regression analysis of gender, serum albumin, glomerular filtration rate, serum creatinine, and 24-hour proteinuria with the overall clinical remission rate.

Variables	Studies(n)	Included patients	Meta—regression			
			coefficient	(95%CI)		P value
Male(%)	12	222	0.014	-0.044	0.015	0.343
serum albumin(g/L)	11	216	-0.101	-0.265	0.062	0.229
eGFR(mL/min/1.73 m^2^)	7	158	-0.011	-0.047	0.026	0.574
serum creatinine(µmol/L)	12	222	0.0002	-0.028	0.029	0.99
24-hour proteinuria (g/d)	9	174	-0.055	-0.247	0.137	0.674

##### 3.4.1.2 CR rate

**Figure 3:**
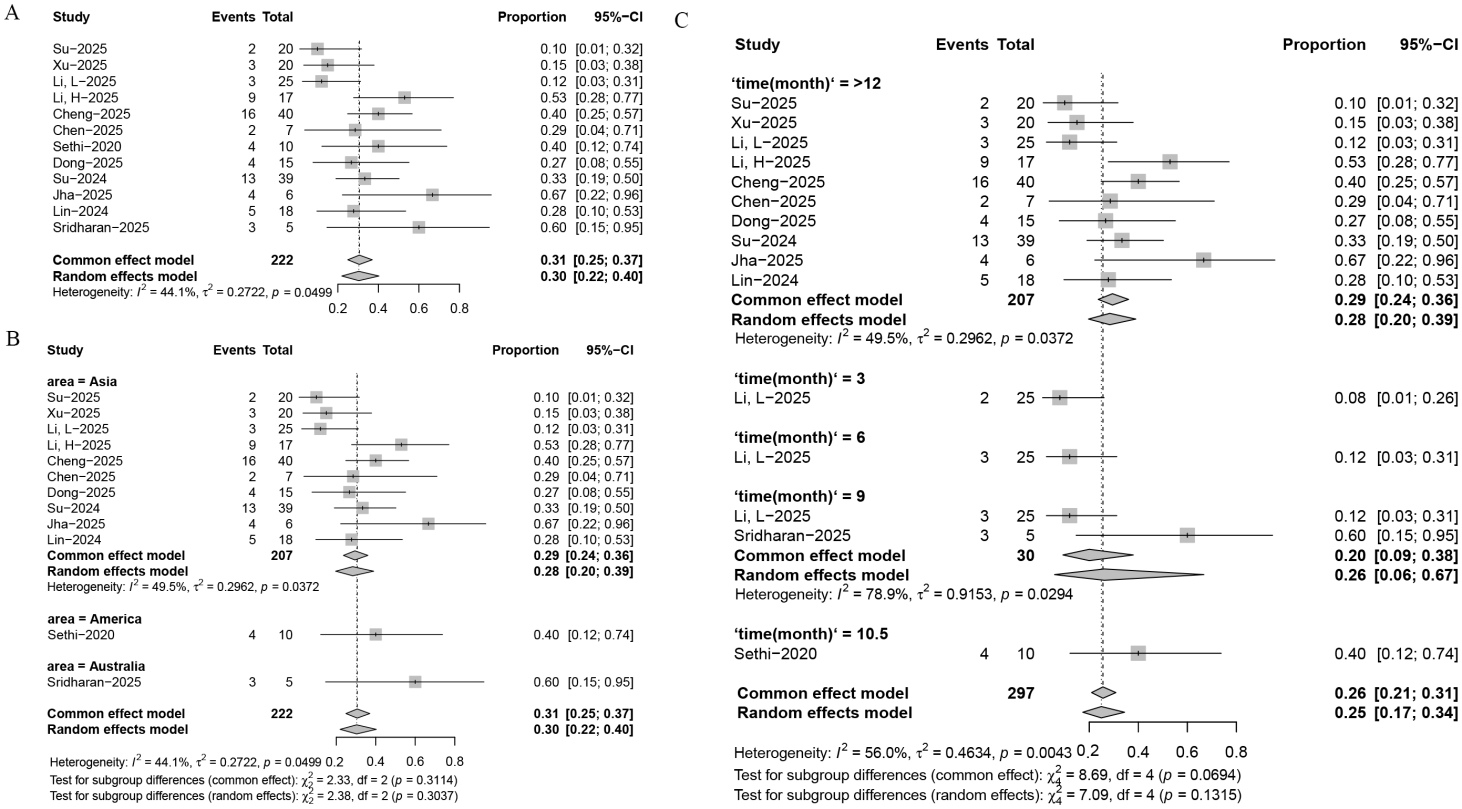
**(A)** Forest plot of the complete clinical remission rate; **(B)** Forest plot of the subgroup analysis of area for the complete clinical remission rate; **(C)** Forest plot of the subgroup analysis of follow-up time for the complete clinical remission rate.

Twelve investigations examined the CR rate. Heterogeneity was low (I² = 44.1%, p = 0.050), and a fixed-effects model was adopted. The pooled CR rate was 31% (95% CI: 25% to 37%; **Figure 3A**). Subgroup analysis by region revealed a CR rate of 29% (24% to 36%) for Asia. For single studies from America and Australia, only the individual study results were presented: 40% (10) and 60% (14), respectively (**Figure 3B**). Because only one study was available from each of these regions, the samples lacked representativeness, and the findings could not be generalized to the broader American or Australian populations. Pooled CR rate according to follow-up duration was as follows: 3 months, 8.0% (1.0% to 26%); 6 months, 12% (3% to 31%); 9 months, 26% (6% to 67%); 10.5 months, 40% (12% to 74%); ≥ 12 months, 29% (24% to 36%; **Figure 3C**). Regression analyses examining associations of CR rate with sex and pre-treatment laboratory parameters (24hUP, SA, SCr, eGFR) demonstrated no marked associations (**Table 4**). Given the modest number of contributing studies, statistical power was limited. The absence of significant associations observed in these regression analyses should therefore be regarded as exploratory and lacking confirmatory inferential value.

**Table 4:** Regression analysis of gender, serum albumin, glomerular filtration rate, serum creatinine, and 24-hour proteinuria with the complete clinical remission rate.

Variables	Studies(n)	Included patients	Meta—regression			
			coefficient	(95%CI)		P value
Male(%)	12	222	-0.314	-0.044	0.017	0.402
serum albumin(g/L)	11	216	-0.990	0.212	0.057	0.211
eGFR(mL/min/1.73 m^2^)	7	158	-0.015	-0.05	0.018	0.364
serum creatinine(µmol/L)	12	222	0.023	-0.004	0.050	0.095
24-hour proteinuria (g/d)	9	174	0.055	-0.247	0.137	0.574

##### 3.4.1.3 PR rate

**Figure 4:**
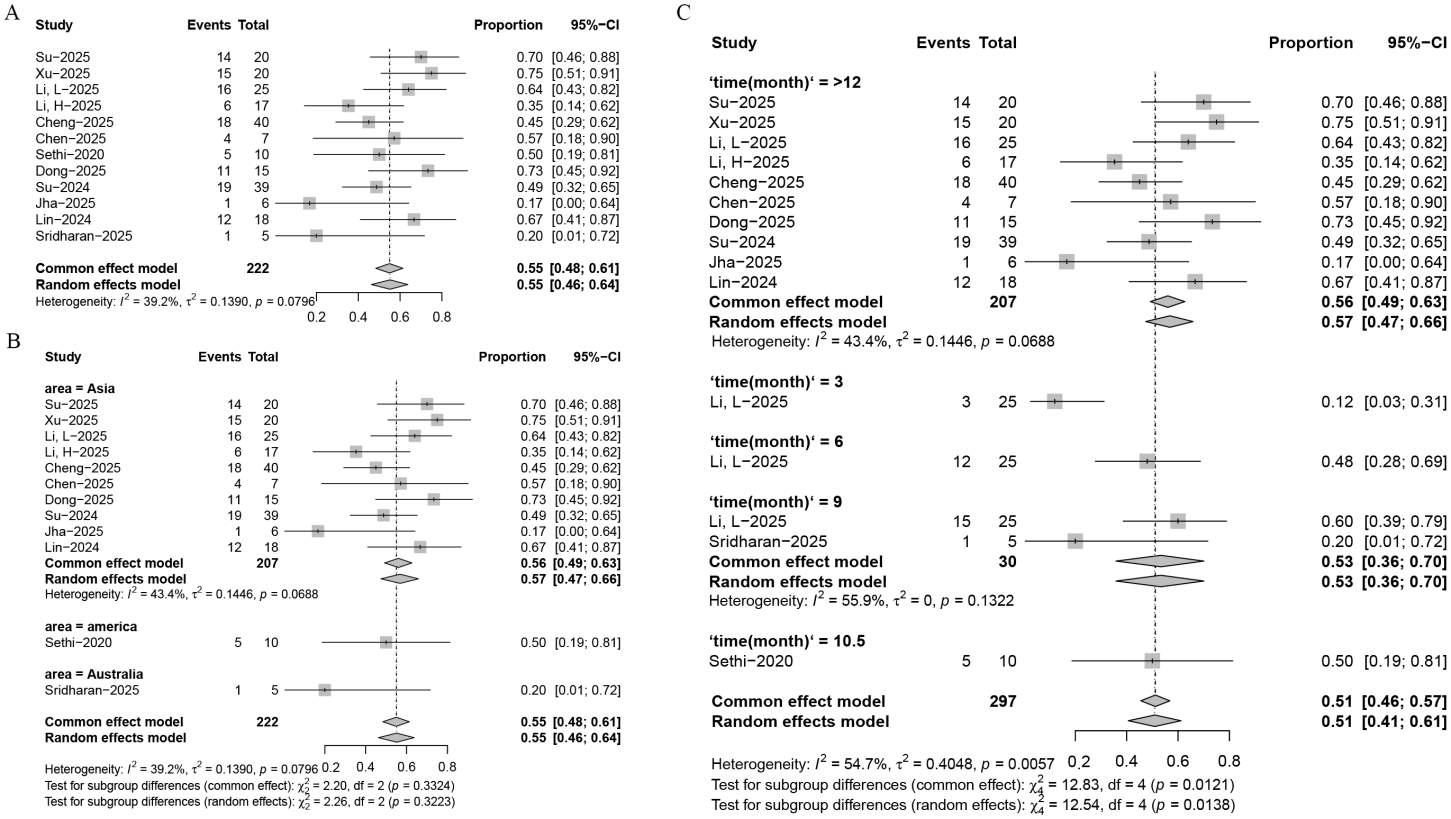
**(A)** Forest plot of the partial clinical remission rate; **(B)** Forest plot of the subgroup analysis of area for the partial clinical remission rate; **(C)** Forest plot of the subgroup analysis of follow-up time for the partial clinical remission rate.

Twelve investigations reported the PR rate. Heterogeneity was low (I² = 39.2%, p = 0.080), and a fixed-effects model was thus applied. The pooled PR rate was 55% (95% CI: 48% to 61%; **Figure 4A**). Subgroup analysis by region yielded a rate of 56% (49% to 63%) in Asia. For single studies from America and Australia, only the individual study results were presented: 50% (10) and 20% (14), respectively (**Figure 4B**). Because only one study was available from each of these regions, the samples lacked representativeness, and the findings could not be generalized to the broader American or Australian populations. Pooled PR rate stratified by follow-up duration was: 3 months, 12% (3% to 31%); 6 months, 48% (28% to 69%); 9 months, 53% (36% to 70%); 10.5 months, 50% (19% to 81%); ≥ 12 months, 56% (49% to 63%; **Figure 4C**). Regression analyses evaluating associations of PR rate with sex and pre-treatment laboratory parameters (24hUP, SA, SCr, eGFR) detected no marked associations (**Table 5**). Given the modest number of contributing studies, statistical power was limited. The absence of significant associations observed in these regression analyses should therefore be regarded as exploratory and lacking confirmatory inferential value.

**Table 5:** Regression analysis of gender, serum albumin, glomerular filtration rate, serum creatinine, and 24-hour proteinuria with the partial clinical remission rate.

Variables	Studies(n)	Included patients	Meta—regression			
			coefficient	(95%CI)		P value
Male(%)	12	222	0.005	-0.022	0.032	0.719
serum albumin(g/L)	11	216	0.039	-0.098	0.178	0.574
eGFR(mL/min/1.73 m^2^)	7	158	0.011	-0.174	0.038	0.457
serum creatinine (µmol/L)	12	222	-0.171	-0.038	0.004	0.109
24-hour proteinuria(g/d)	9	174	-0.551	-0.247	0.137	0.574

##### 3.4.1.4 IR rate

**Figure 5:**
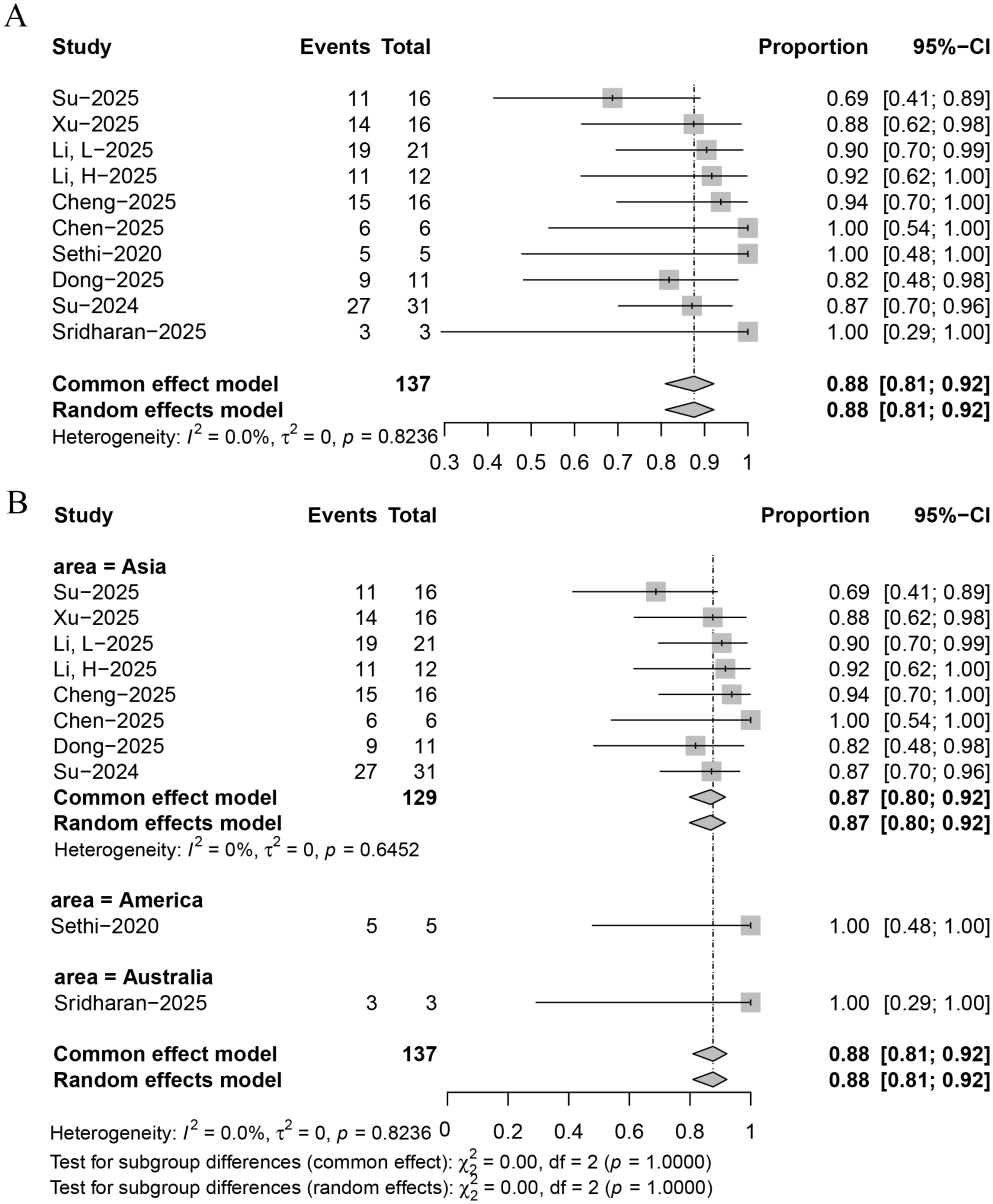
**(A)** Forest plot of the immune remission rate; **(B)** Forest plot of the subgroup analysis of area for the immune remission rate.

Ten investigations assessed the IR rate. Heterogeneity was low (I² = 0.0%, p = 0.824), and a fixed-effects model was utilized. The pooled IR rate was 88% (95% CI: 81% to 92%; **Figure 5A**). Subgroup analysis by region revealed an IR rate of 87% (80% to 92%) in Asia. For single studies from America and Australia, only the individual study results were presented: 100% (10) and 100% (14), respectively (**Figure 5B**). Because only one study was available from each of these regions, the samples lacked representativeness, and the findings could not be generalized to the broader American or Australian populations. Regression analyses examining associations of the IR rate with sex and pre-treatment titer of anti-PLA2R antibody indicated no marked associations (**Table 6**). Given the modest number of contributing studies, statistical power was limited. The absence of significant associations observed in these regression analyses should therefore be regarded as exploratory and lacking confirmatory inferential value.

**Table 6:** Regression analysis of gender and the titer of anti-PLA2R antibody with the immune remission rate.

Variables	Studies(n)	Included patients	Meta—regression			
			coefficient	(95%CI)		P value
Male(%)	12	222	-0.015	-0.044	0.015	0.343
anti-PLA2R antibody(RU/mL)	12	222	0.005	-0.006	0.015	0.363

#### 3.4.2 Laboratory parameters

##### 3.4.2.1 Proteinuria

**Figure 6:**
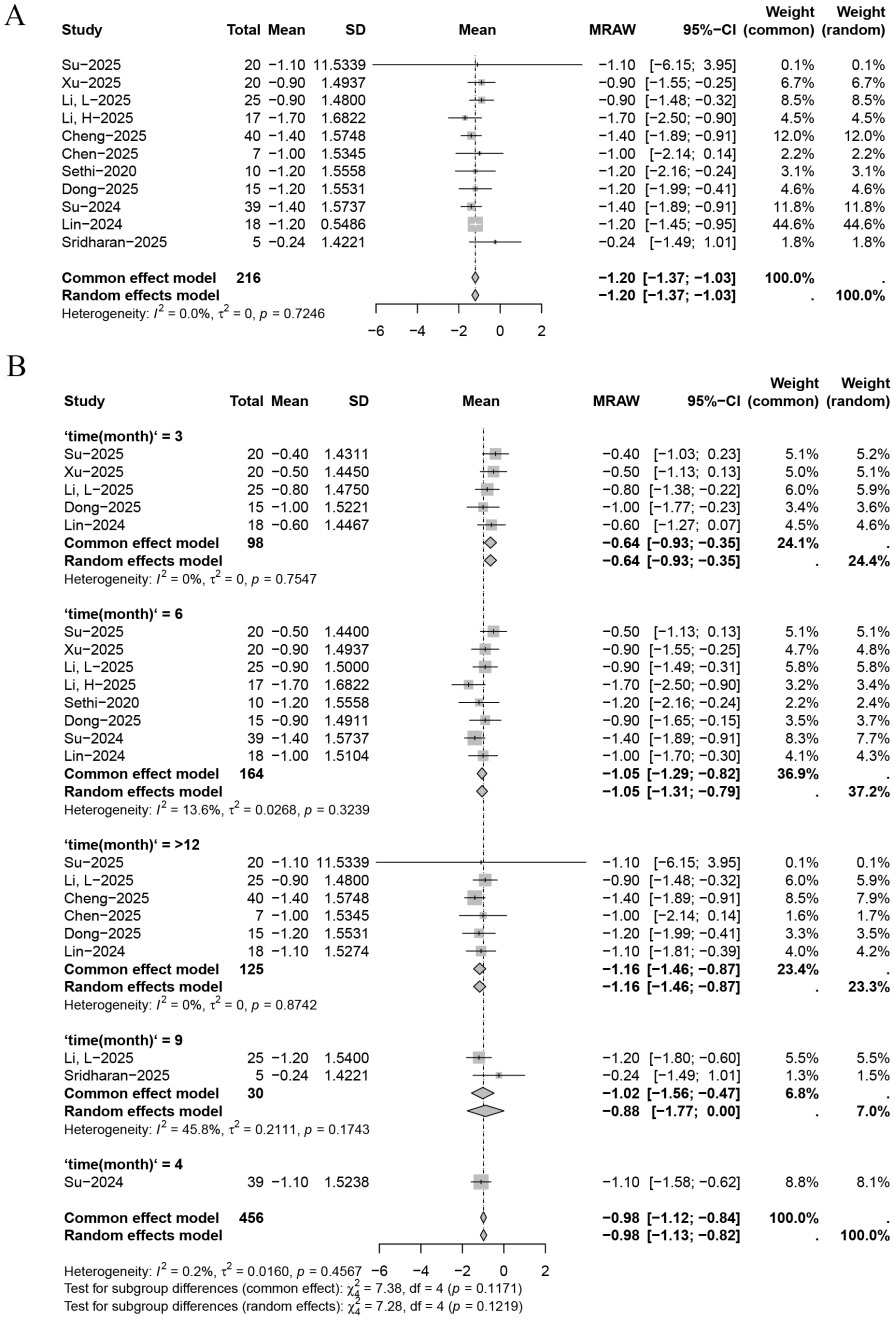
**(A)** Forest plot of proteinuria; **(B)** Forest plot of the subgroup analysis of follow-up time for proteinuria.

Eleven investigations provided pre- and post-treatment proteinuria levels. Heterogeneity was low (I² = 0.0%, p = 0.725), and a fixed-effects model was thus adopted. Obinutuzumab therapy was associated with a reduction in proteinuria (SMD = -1.2; 95% CI: -1.37 to -1.03; **Figure 6A**), suggesting a large clinical effect of obinutuzumab on lowering proteinuria. Subgroup analysis by follow-up duration yielded pooled SMDs of -0.64 (95% CI: -0.93 to -0.35) at 3 months, -1.1 (-1.58 to -0.62) at 4 months, -1.05 (-1.29 to -0.82) at 6 months, -1.02 (-1.56 to -0.47) at 9 months, and -1.16 (-1.46 to -0.87) at ≥ 12 months (**Figure 6B**).

##### 3.4.2.2 SA

**Figure 7:**
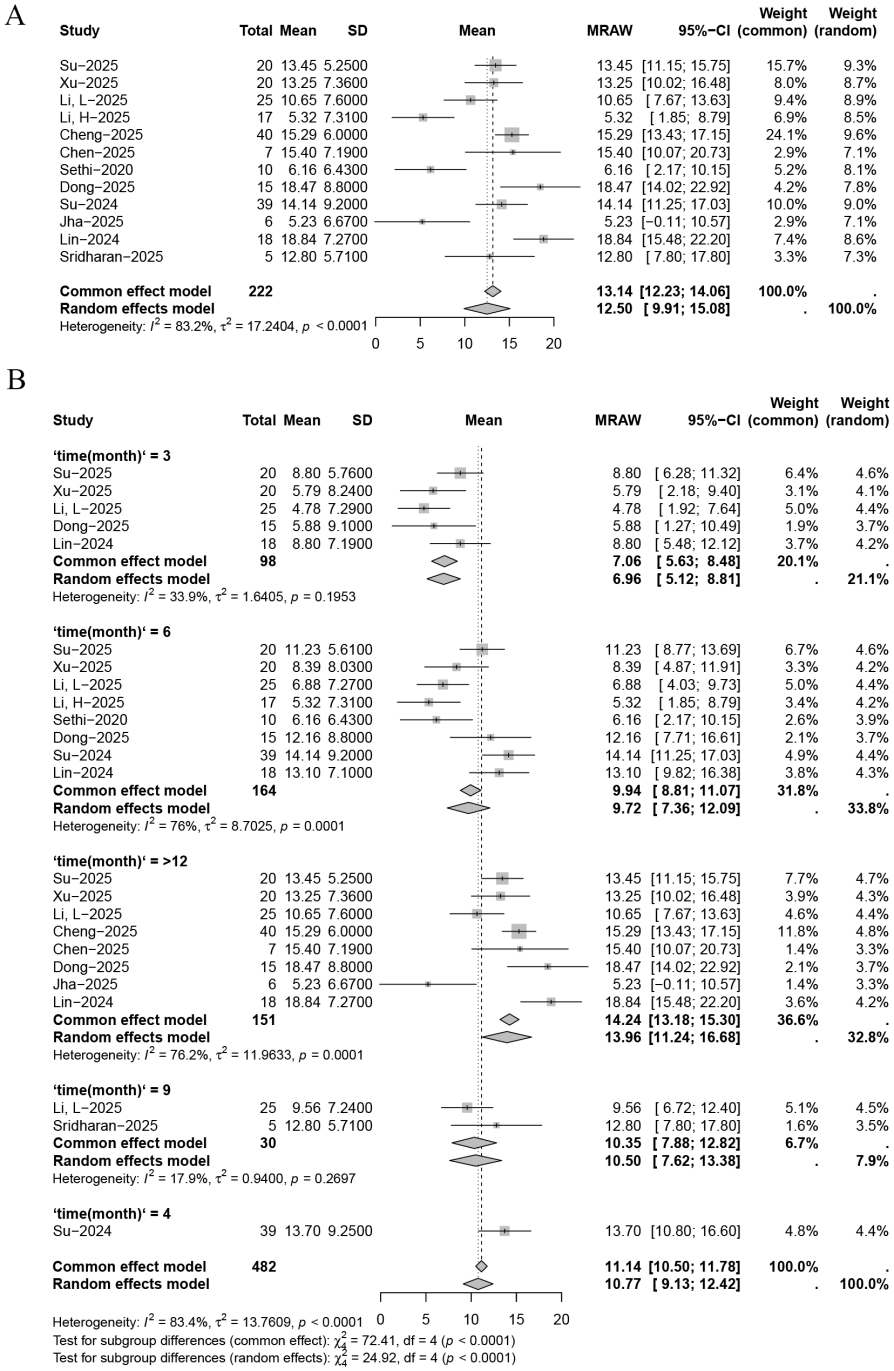
**(A)** Forest plot of serum albumin; **(B)** Forest plot of the subgroup analysis of follow-up time for serum albumin.

Twelve investigations reported pre- and post-treatment measurements of SA. Substantial heterogeneity was noted (I² = 83.2%, p < 0.001), and a random-effects model was applied. Treatment with obinutuzumab was related to an elevation in SA (MD = 12.5 g/L; 95% CI: 9.91 to 15.08; **Figure 7A**). To explore sources of heterogeneity, subgroup analysis by follow-up duration was performed. The pooled MDs were 7.06 (95% CI: 5.63 to 8.48) at 3 months, 13.7 (10.80 to 16.60) at 4 months, 9.72 (7.36 to 12.09) at 6 months, 10.35 (7.88 to 12.82) at 9 months, and 13.96 (11.24 to 16.68) at ≥ 12 months (**Figure 7B**). Follow-up duration did not fully account for the observed heterogeneity.

##### 3.4.2.3 SCr

**Figure 8:**
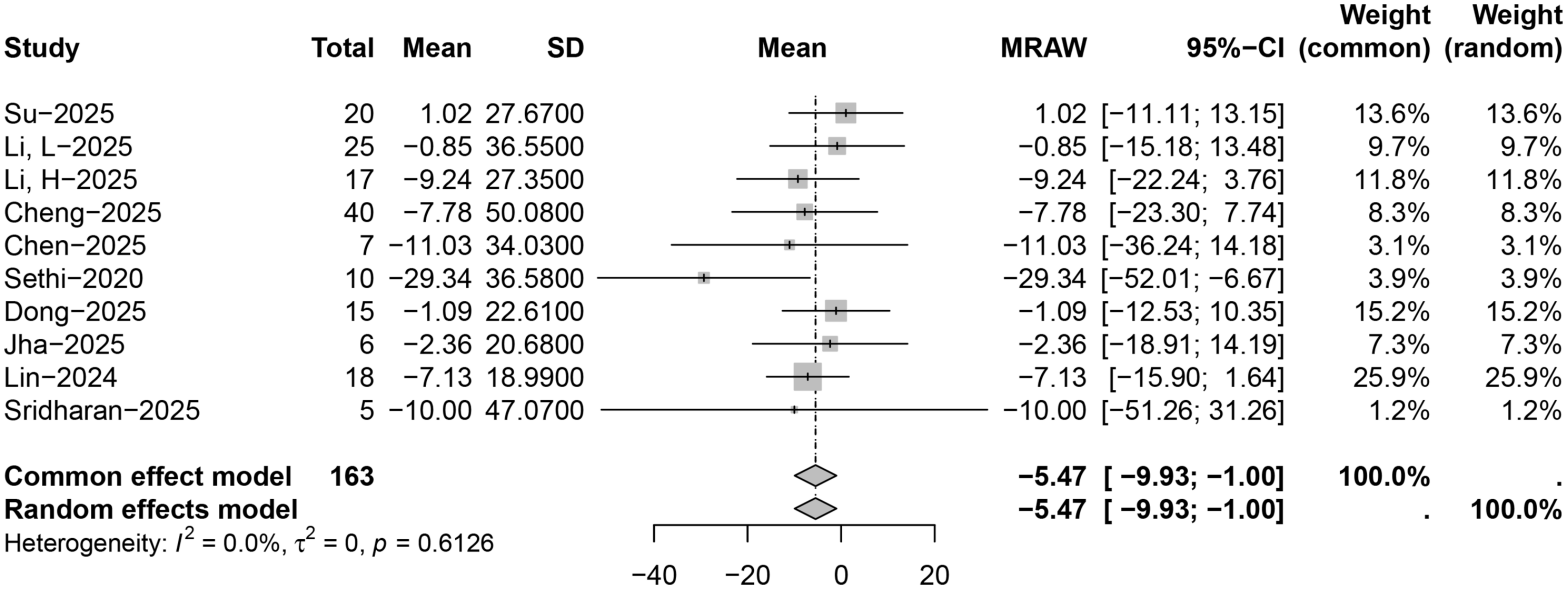
Forest plot of serum creatinine.

Ten investigations provided pre- and post-treatment data on SCr. Heterogeneity was low (I² = 0.0%, p = 0.613), and a fixed-effects model was employed. Obinutuzumab therapy was associated with a reduction in SCr (MD = -5.47 µmol/L; 95% CI: -9.93 to -1; **Figure 8**). Although statistically significant, the magnitude of the reduction in SCr may be clinically negligible.

##### 3.4.2.4 eGFR

**Figure 9:**
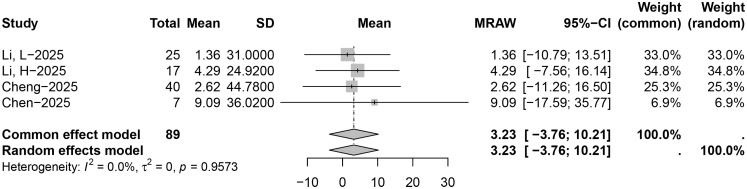
Forest plot of golomeruar filtration rate.

Four investigations reported pre- and post-treatment values of eGFR. Heterogeneity was low (I² = 0.0%, p = 0.957), and a fixed-effects model was utilized. Treatment with obinutuzumab did not improve eGFR (MD = 3.23 mL/min/1.73m²; 95% CI: -3.76 to 10.21; **Figure 9**).

#### 3.4.3 Safety outcomes

**Figure 10:**
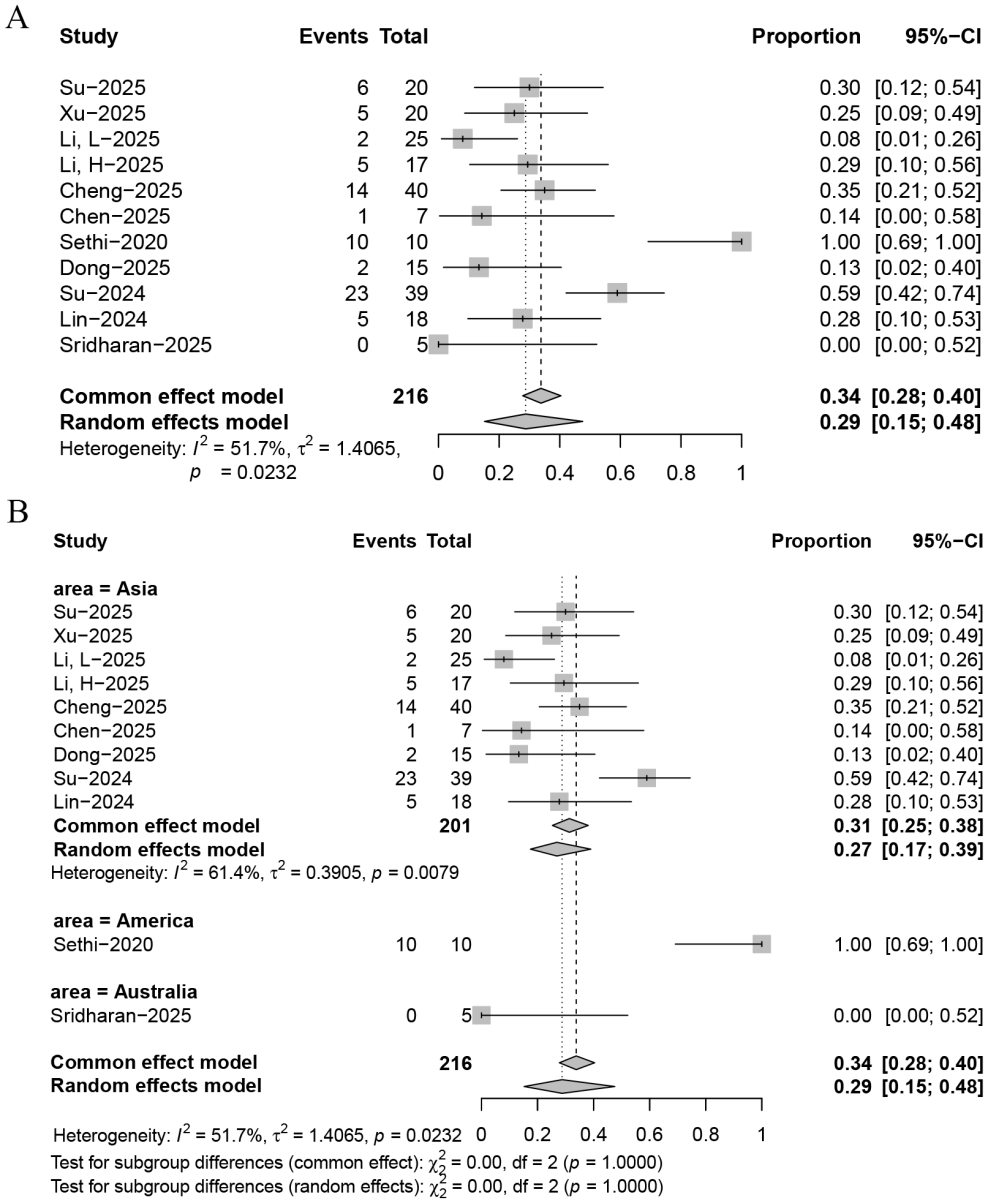
**(A)** Forest plot of the overall Adverse Event Incidence; **(B)** Forest plot of the subgroup analysis of area for the overall Adverse Event Incidence.

Twelve investigations reported the overall incidence of AEs. Heterogeneity was high across studies (I² = 51.7%, p = 0.023), and a random-effects model was applied. The pooled overall incidence of AEs was 29% (95% CI: 15% to 48%; **Figure 10A**). Subgroup analysis was implemented to detect sources of heterogeneity. Following stratification by region, the overall incidence of AEs was 27% (17% to 39%) in Asia, 100% in the single American investigation (10), and 0% in the single Australian study (14) (**Figure 10B**). This suggested that region was not a source of heterogeneity. Specific incidences of AEswere as follows: cytomegalovirus viremia, 13% (3% to 41%); pneumonia, rash, and pruritus, 11% (6% to 18%); pyrexia and leukopenia, 8% (4% to 15%); infection of upper respiratory tract, 7% (3% to 15%); infection of urinary tract, 6% (2% to 20%); (**Supplementary File 2**). Additionally, because certain AEs—including death, soft tissue infection, hepaticdysfunction, biliary tract infection, and tinnitus—were sporadic and exhibited extremely low incidence, pooling their incidence rates would likely produce unstable summary estimates. Consequently, only the aggregated counts of each rare AE reported in the included studies were summarized (**Supplementary File 5**).

### 3.5 Sensitivity analysis and publication bias

**Figure 11:**
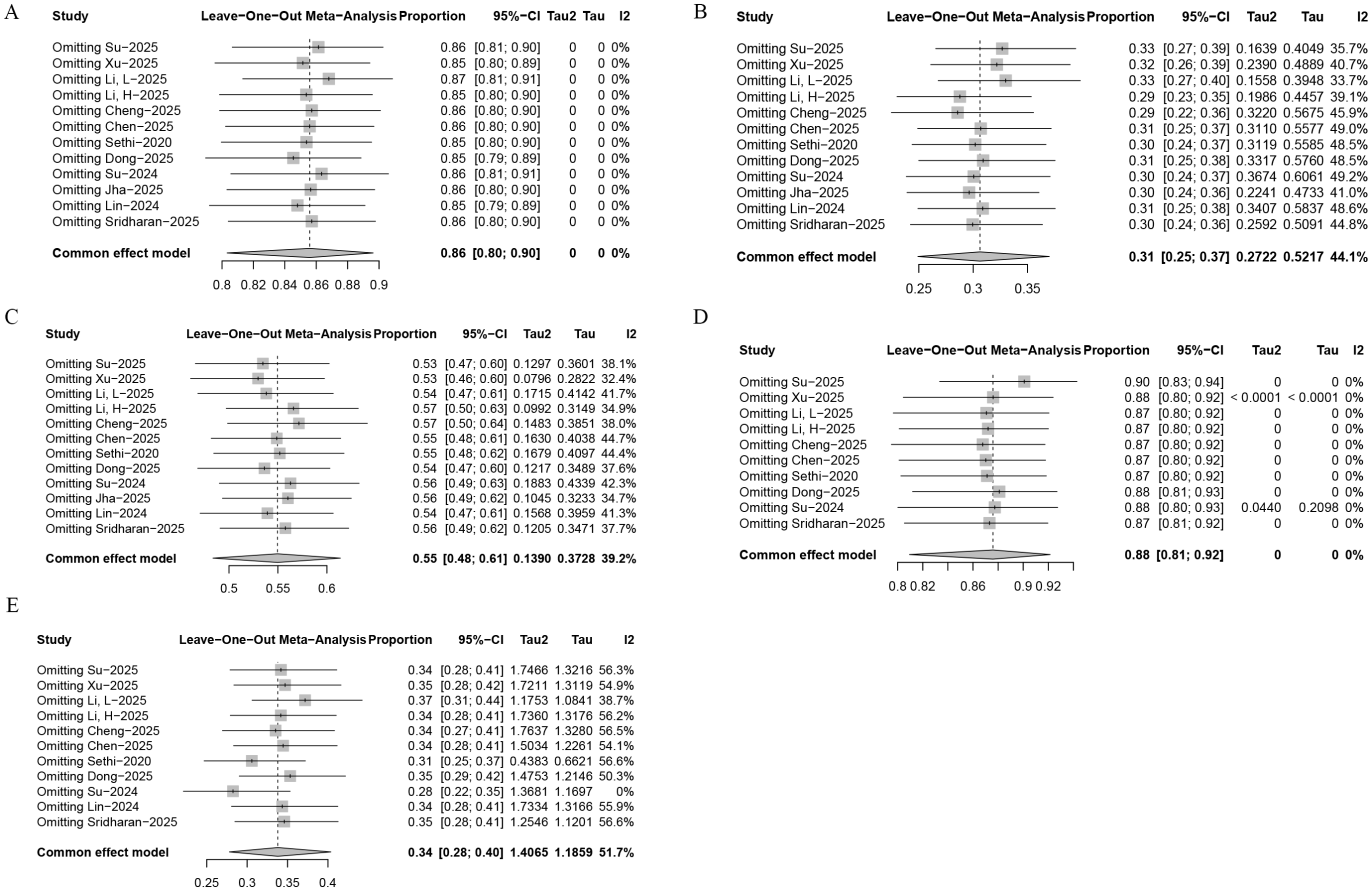
**(A)** Sensitivity analysis of the overall clinical remission rate; **(B)** Sensitivity analysis of the complete clinical remission rate; **(C)** Sensitivity analysis of the partial clinical remission rate; **(D)** Sensitivity analysis of the immune remission rate; **(E)** Sensitivity analysis of the overall Adverse Event Incidence.

**Figure 12:**
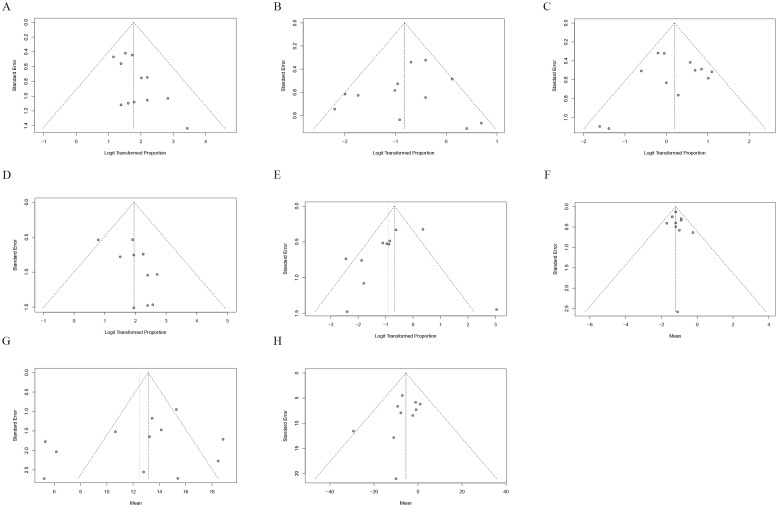
**(A)** Funnel plot of the overall clinical remission rate; **(B)** Funnel plot of the complete clinical remission rate; **(C)** Funnel plot of the partial clinical remission rate; **(D)** Funnel plot of the immune remission rate; **(E)** Funnel plot of the overall Adverse Event Incidence; **(F)** Funnel plot of the proteinuria; **(G)** Funnel plot of the serum albumin; **(H)** Funnel plot of the serum creatinine.

**Figure 13:**
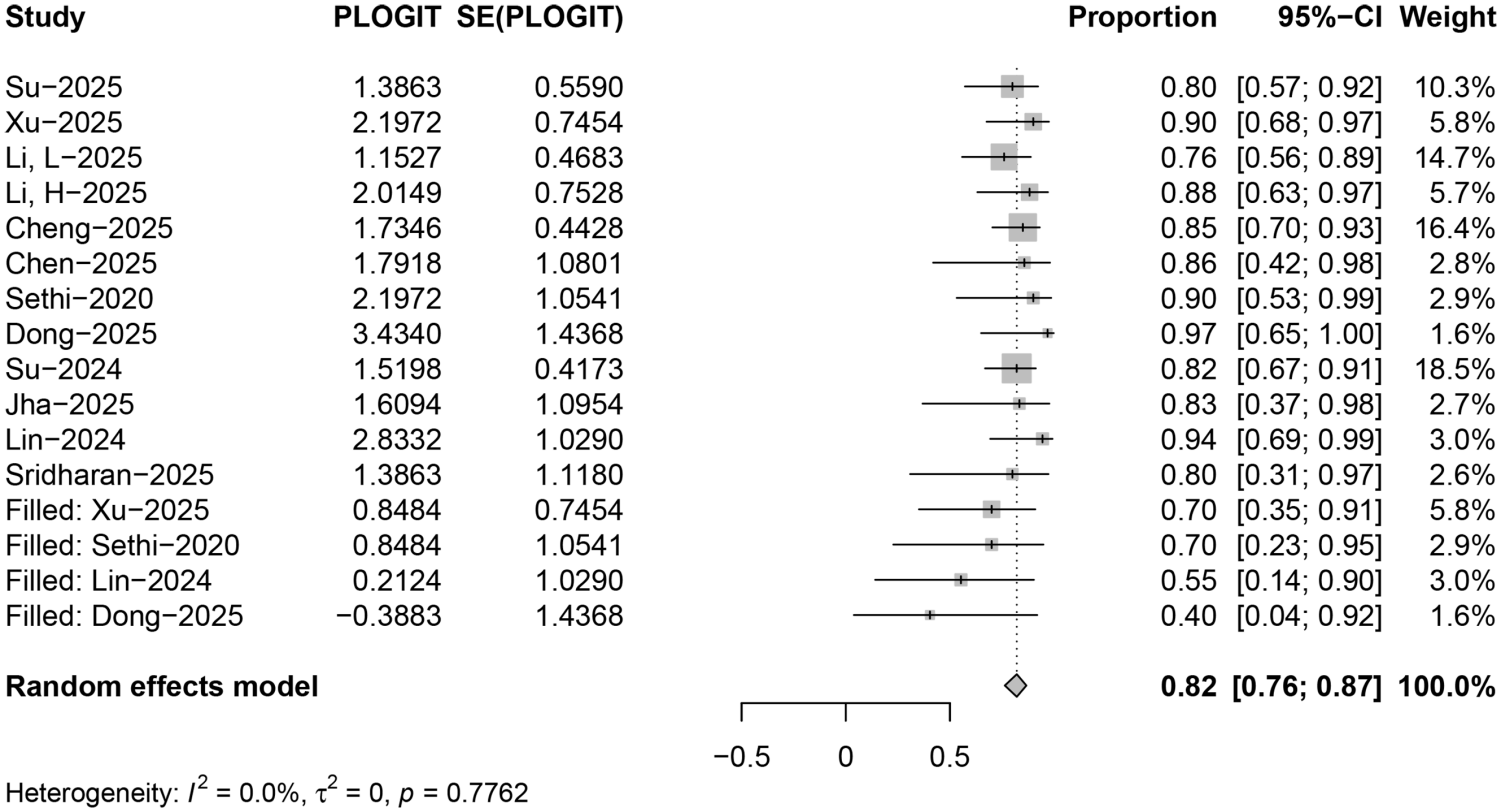
The overall clinical remission rate after the trim-and-fill method.

Sensitivity analyses, conducted by sequential omission of individual investigations, did not materially alter the direction or statistical significance of pooled effect estimates, underscoring the robustness of the findings (**Figures 11A–E** for primary outcomes; **Supplementary File 3** for laboratory parameters and specific AEs). Funnel plots were generated for OR, CR, PR, and IR rates, overall incidence of AEs, and laboratory parameters (pre- to post-treatment differences in proteinuria, SA, and SCr). Apart from the plot for OR rate, all funnel plots exhibited approximate symmetry (**Figures 12A–H**). Assessment of publication bias was not undertaken for specific AEs or for pre- to post-treatment difference in eGFR, owing to the limited number of eligible studies (fewer than ten). Egger’s test indicated no notable publication bias for CR rate (p = 0.618), PR rate (p = 0.744), IR rate (p = 0.072), overall incidence of AEs (p = 0.252), and pre- to post-treatment differences in proteinuria (p = 0.497), SA (p = 0.325), or SCr (p = 0.271). Egger’s test suggested significant publication bias for the OR rate (p = 0.026). Trim-and-fill analysis was implemented, adjusting the OR rate from 86% (95% CI: 80% to 90%) to 82% (76% to 87%; **Figure 13**). This implied a possible slight decrease in OR rate; nonetheless, the adjusted OR rate remained substantial.

## 4 Discussion

The present research constitutes the first single-arm meta-analysis to evaluate the efficacy and safety of obinutuzumab in refractory MN, corroborating its favorable therapeutic profile and considerable promise in this clinical context. Obinutuzumab markedly reduced excretion of proteinuria and SCr while elevating concentrations of SA. The OR rate reached 86%, with the CR rate at 31%, the PR rate at 55%, and the IR rate at 88%. The overall incidence of AEs was 29%. Two publications documented two fatal severe adverse events occurring during the treatment with obinutuzumab —one resulting from severe COVID-19 pneumonia and one attributable to pneumonia-associated respiratory failure. One of the two individuals had undergone eight prior rounds of immunosuppressive therapy before receiving obinutuzumab. This observation underscores that caution is required when managing individuals with an extensive history of immunosuppressive therapy, given the heightened risk of severe infection that may culminate in fatal outcomes.

A network meta-analysis by Zhao et al. (23) reveals pronounced efficacy of obinutuzumab in lupus nephritis, a finding consonant with our results. In the specific domain of refractory MN, the present analysis indicated that 86% of affected individuals achieved clinical remission following obinutuzumab therapy. This aligns closely with outcomes reported in previously published clinical series by Li et al. (18), Cheng et al. (19), and Chen et al. (20), collectively substantiating the marked efficacy of obinutuzumab for refractory MN. The research by Dong et al. (11)reports an OR rate of 100%. This discrepancy may stem from factors including modest sample size, abbreviated follow-up duration, and variation in dosing frequency within that specific investigation. The recently published MAJESTY phase III RCT (24) first demonstrates that obinutuzumab is superior to tacrolimus in treating PMN. This establishes high-level evidence supporting the first-line use of obinutuzumab in treatment-naïve or active PMN. This study represents the first single-arm meta-analysis evaluating the efficacy of obinutuzumab in refractory MN. Regarding comparative efficacy, the pooled OR rate (86%) exceeded the 51% reported in the MAJESTY trial. Such a difference is potentially attributable to statistical methodology (MAJESTY employed a stringent composite strategy in which escape therapy was counted as non-response). Additionally, the present analysis comprised uncontrolled, retrospective single-arm studies and case series, and the OR rate may be overestimated due to selection bias. The pooled CR rate (31%) approximated the CR rate for the obinutuzumab arm in the MAJESTY trial (37%). This suggests that even within a more complex refractory setting, obinutuzumab can induce a substantial proportion of complete remissions, consistent with its potent B-cell depletion effect. In terms of renal protection and safety, the current findings corroborate those of the MAJESTY trial. Both indicate that obinutuzumab primarily stabilizes renal function rather than markedly elevating eGFR. Simultaneously, both studies reported low rates of serious adverse events, and the principal risk involved infections, particularly SARS-CoV-2 infection and associated pneumonia. Furthermore, a recent systematic review by Atay et al. (25) also synthesizes data on obinutuzumab for MN (89 individuals, 64% with refractory disease). It reports OR and IR rates of 83% and 88.7%, respectively, closely aligning with the present results (OR rate: 86%; IR rate: 88%). Their subgroup analysis, however, indicates a CR rate of 23.5% in refractory cases, somewhat lower than the 31% observed herein. This may reflect differences in dosing frequency and cumulative dose of obinutuzumab administered to individuals with refractory MN. Both pooled analyses are dominated by data from China (72% of individuals in the study by Atay et al.; 90.5% in this study), representing a geographic concentration. Large-scale, multicenter, cross-ethnic RCTs are needed for further validation.

MN is considered an autoimmune disorder. This provides a mechanistic rationale for B-cell-directed therapy. Obinutuzumab is a humanized type II anti-CD20 monoclonal antibody. The Fc fragment of obinutuzumab has undergone glycoengineering. This structural modification augments its affinity for FcγRIIIA receptors on B cells, thereby potentiating binding and recruitment of effector cells. Relative to type I anti-CD20 monoclonal antibodies such as RTX, obinutuzumab demonstrates heightened binding affinity for immunoglobulin receptors, enhanced antibody-dependent cellular cytotoxicity, superior direct B-cell cytotoxicity, diminished reliance on complement-dependent cytotoxicity, and decreased risk of immunogenicity (10, 26). Obinutuzumab also induces lysosome-mediated direct cell death and mitigates dependence on elevated levels of B-cell activating factors, which may facilitate more effective depletion of memory B-cell populations. Consequently, memory B cells exhibit reduced resistance to obinutuzumab relative to RTX (27). Furthermore, owing to its target-binding characteristics, drug clearance of obinutuzumab is 4.3-fold lower than that of RTX, the half-maximal effective concentration is 15-fold lower, and the cellular killing coefficient is 4.7-fold greater. Obinutuzumab thus exhibits prolonged *in vivo* activity, sustained therapeutic effect, and more profound B-lymphocyte depletion (28), thereby conferring marked efficacy in refractory MN.

The current meta-analysis furnishes clinicians with an evidence-based foundation for managing refractory MN. Its findings indicate that obinutuzumab may yield higher remission rates, thereby slowing the progression of renal diseases, improving quality of life, and enhancing long-term outcomes.

Several limitations should be acknowledged. First, all eligible investigations were single-arm, retrospective, single-center studies lacking comparator arms. Incomplete data collection and variable and occasionally abbreviated follow-up durations may collectively compromise the precision of effect estimates. Second, substantial heterogeneity was detected in the analyses of SA and the overall incidence of AEs. Despite subgroup analyses, sources of heterogeneity remained unidentified. Plausible contributors—including precise doses of drug, dosing frequency, and details of prior immunosuppressive regimens—could not be extracted with sufficient granularity for further exploration. Third, the accuracy of AE assessment may be influenced by the limited number of contributing publications and modest sample sizes per study. Fourth, because of the small number of included studies, meta-regression and subgroup analyses may have insufficient statistical power. In the subgroup analysis by region, only one study from America and Australia each was available, the representativeness of samples was inadequate, and the findings could not be generalized to the broader populations of these regions. Under these conditions, the absence of statistically significant differences between subgroups and the null findings from regression analyses may reflect inadequate power rather than true biological equivalence or lack of association. These results should therefore not be interpreted as definitive conclusions. Fifth, standards could not be unified because of the heterogeneity in antecedent immunosuppressive regimens. The generalizability across diverse ethnic cohorts is constrained due to the predominance of East Asian populations (90.5% of individuals were from China). The pathogenesis of MN exhibits marked ethnic genetic heterogeneity. A cross-ancestry genome-wide association study (GWAS) demonstrates that the principal risk allele is HLA-DRB1*1501 (odds ratio = 3.81) in East Asian populations and DQA1*0501 (odds ratio = 2.88) in European populations. GWAS-explained disease risk accounts for 32% in East Asians versus 25% in Europeans (29). Moreover, the choice of immunosuppressive regimens and standards of supportive care also vary across global regions. This geographic concentration thus constitutes an important limitation. These factors may have introduced bias into the findings. The aforementioned limitations collectively underscore the imperative for future large-scale, multicenter, cross-ethnic, prospective RCTs with extended follow-up to more comprehensively validate the efficacy and safety of obinutuzumab in refractory MN. Such prospective research would facilitate more detailed data acquisition, thorough safety evaluation, and refined subgroup analyses stratified by variables such as ethnicity, cumulative dose of obinutuzumab, and prior therapeutic exposure. Extended and harmonized follow-up protocols would further enable robust appraisal of long-term therapeutic durability.

## 5 Conclusion

The present meta-analysis demonstrates that obinutuzumab exhibits marked therapeutic efficacy in individuals with refractory MN, accompanied by an OR rate of 86% and a low incidence of severe adverse events. Obinutuzumab could have a therapeutic benefit for refractory MN. Nonetheless, all raw data included in this meta-analysis were derived from uncontrolled retrospective single-arm trials and case series. Given factors such as selection bias, the remission rate may be overestimated. The therapeutic effectiveness and safety profile of obinutuzumab should be further validated in large-scale, multicenter, cross-ethnic, prospective RCTs.

## Data Availability

The original contributions presented in the study are included in the article/**Supplementary Material**. Further inquiries can be directed to the corresponding author.

## Supplementary material

Supplementary File 1 retrieval formula.

Supplementary File 2 Forest plot of the incidence of specific adverse events.

Supplementary File 3 Sensitivity analysis of the incidence of specific adverse events.

Supplementary File 4 The definitions of clinical remission and immune remission.

Supplementary File 5 Rare adverse events.
