# Supplementary material for: Efficacy and safety of obinutuzumab in refractory membranous nephropathy: a single-arm meta-analysis
Source: Front Immunol. 2026 Jul 21;17:1880420. doi: 10.3389/fimmu.2026.1880420 (PMC13433739; doi:10.3389/fimmu.2026.1880420)
Supplement: Supplementary file 1 [file Table1.docx]

**Supplementary materials**

**Supplementary File 1** retrieval formula.

| Pubmed | |
| --- | --- |
| # | Query |
| 1 | Glomerulonephritis, Membranous[MeSH Terms] |
| 2 | "extramembranous glomerulonephritis"[Title/Abstract] OR "extramembranous glomerulopathy"[Title/Abstract] OR "Heymann Nephritis"[Title/Abstract] OR "membranous glomerulitis"[Title/Abstract] OR "Membranous Glomerulonephritides"[Title/Abstract] OR "membranous glomerulonephritis"[Title/Abstract] OR "Membranous Glomerulonephropathy"[Title/Abstract] OR "membranous glomerulopathy"[Title/Abstract] OR "membranous nephritis"[Title/Abstract] OR "membranous nephropathy"[Title/Abstract] |
| 3 | #2 OR #1 |
| 4 | obinutuzumab [Supplementary Concept] |
| 5 | "afutuzumab"[Title/Abstract] OR "ga 101"[Title/Abstract] OR "ga101"[Title/Abstract] OR "gazyva*"[Title/Abstract] OR "obinutuzumab"[Title/Abstract] OR "r 7159"[Title/Abstract] OR "r7159"[Title/Abstract] OR "rg 7159"[Title/Abstract] OR "rg7159"[Title/Abstract] OR "ro 5072759"[Title/Abstract] OR "ro5072759"[Title/Abstract] |
| 6 | #4 OR #5 |
| 7 | #3 AND #6 |

| Embase | |
| --- | --- |
| # | Query |
| 1 | 'membranous glomerulonephritis'/exp |
| 2 | 'extramembranous glomerulonephritis':ti,ab,kw OR 'extramembranous glomerulopathy':ti,ab,kw OR 'heymann nephritis':ti,ab,kw OR 'membranous glomerulitis':ti,ab,kw OR 'membranous glomerulonephritides':ti,ab,kw OR 'membranous glomerulonephritis':ti,ab,kw OR 'membranous glomerulonephropathy':ti,ab,kw OR 'membranous glomerulopathy':ti,ab,kw OR 'membranous nephritis':ti,ab,kw OR 'membranous nephropathy':ti,ab,kw |
| 3 | #1 OR #2 |
| 4 | 'obinutuzumab'/exp |
| 5 | 'afutuzumab':ti,ab,kw OR 'ga 101':ti,ab,kw OR 'ga101':ti,ab,kw OR 'gazyva*':ti,ab,kw OR 'obinutuzumab':ti,ab,kw OR 'r 7159':ti,ab,kw OR 'r7159':ti,ab,kw OR 'rg 7159':ti,ab,kw OR 'rg7159':ti,ab,kw OR 'ro 5072759':ti,ab,kw OR 'ro5072759':ti,ab,kw |
| 6 | #4 OR #5 |
| 7 | #3 AND #6 |

| Cochrane Library | |
| --- | --- |
| # | Query |
| 1 | MeSH descriptor: [Glomerulonephritis, Membranous] explode all trees |
| 2 | ('extramembranous glomerulonephritis' OR 'extramembranous glomerulopathy' OR 'Heymann Nephritis' OR 'membranous glomerulitis' OR 'Membranous Glomerulonephritides' OR 'membranous glomerulonephritis' OR 'Membranous Glomerulonephropathy' OR 'membranous glomerulopathy' OR 'membranous nephritis' OR 'membranous nephropathy'):ti,ab,kw |
| 3 | #1 OR #2 |
| 4 | ('afutuzumab' OR 'ga 101' OR 'ga101' OR 'gazyva*' OR 'obinutuzumab' OR 'r 7159' OR 'r7159' OR 'rg 7159' OR 'rg7159' OR 'ro 5072759' OR 'ro5072759'):ti,ab,kw |
| 5 | #3 and #4 |

| Web of Science | |
| --- | --- |
| # | Query |
| 1 | TS=((extramembranous glomerulonephritis) OR (extramembranous glomerulopathy) OR (Heymann Nephritis) OR (membranous glomerulitis) OR (Membranous Glomerulonephritides) OR (membranous glomerulonephritis) OR (Membranous Glomerulonephropathy) OR (membranous glomerulopathy) OR (membranous nephritis) OR (membranous nephropathy)) |
| 2 | TS=((afutuzumab) OR (ga 101) OR (ga101) OR (gazyva*) OR (obinutuzumab) OR (r 7159) OR (r7159) OR (rg 7159) OR (rg7159) OR (ro 5072759) OR (ro5072759)) |
| 3 | #43 AND #42 |

**Supplementary File 2** Forest plot of the incidence of specific adverse events.

A: Forest plot of the incidence of cytomegalovirus viremia

B: Forest plot of the incidence of pneumonia

C: Forest plot of the incidence of rash and pruritus

D: Forest plot of the incidence of pyrexia

E: Forest plot of the incidence of leukopenia

F: Forest plot of infection of upper respiratory tract

G: Forest plot of infection of urinary tract

**Supplementary File 3** Sensitivity analysis of the incidence of specific adverse events.

A: Sensitivity analysis of the incidence of the cytomegalovirus viremia

B: Sensitivity analysis of the incidence of pneumonia

C:Sensitivity analysis of the incidence of rash and pruritus

D: Sensitivity analysis of the incidence of pyrexia

E: Sensitivity analysis of the incidence of leukopenia

F: Sensitivity analysis of infection of upper respiratory tract

G: Sensitivity analysis of infection of urinary tract

H: Sensitivity analysis of pre- to post-treatment differences in proteinuria

I: Sensitivity analysis of pre-to post-treatment differences in serum albumin

GJ Sensitivity analysis of pre- to post-treatment differences in SCr

K: Sensitivity analysis of pre- to post-treatment differences in eGFR

**Supplementary File 4** The definitions of clinical remission and immune remission.

| id | author | year | country | design | Sample size | Definition of immunologic remission | Definition of clinical remission | Dosing regimen |
| --- | --- | --- | --- | --- | --- | --- | --- | --- |
| 1 | Su | 2025 | China | Single-arm clinical trail | 20 | Immunologic remission defined as serum anti-PLA2R antibody titer < 2 RU/mL | Per 2021 KDIGO guidelines | Patients initially received two 1 g doses of obinutuzumab administered 2 weeks apart. At 6 months, five patients with urinary protein exceeding 3.5 g/day received additional infusion of obinutuzumab at 1–2 g. Within 6 months, four patients exhibited B-cell reconstitution and received an additional 1–2 g obinutuzumab during the sixth month (nine patients total received supplemental obinutuzumab at month 6) |
| 2 | Xu | 2025 | China | Retrospective cohort study | 20 | Immunologic remission defined as serum anti-PLA2R antibody titer < 2 RU/mL | Per 2021 KDIGO guidelines | Obinutuzumab administered intravenously at a dose of 1 g, given in 1–2 administrations with at least a 2-week interval between doses |
| 3 | Li, L | 2025 | China | Single-arm clinical trail | 25 | Immunologic remission defined as serum anti-PLA2R antibody titer < 2 RU/mL | Complete remission: UPCR < 300 mg/g, no deterioration or progression of renal function. Partial remission: UPCR 300–3500 mg/g with ≥ 50% reduction from peak level, accompanied by normalization or elevation of serum albumin, or stable serum creatinine with < 30% increase from baseline | All patients received a 1000 mg per-dose regimen. Three patients received 1000 mg obinutuzumab infusion, 12 received 2000 mg, 8 received 3000 mg, and 2 received 4000 mg |
| 4 | Li, H | 2025 | China | Single-arm clinical trail | 17 | Immunologic remission defined as serum anti-PLA2R antibody titer < 2 RU/mL | Complete remission (CR) defined as urinary protein excretion < 0.3 g/day, normal serum albumin concentration, and stable renal function. Partial remission (PR) defined as urinary protein excretion < 3.5 g/day with ≥ 50% reduction from baseline, accompanied by improvement or normalization of serum albumin concentration, and stable GFR | Patients received two 1000 mg doses of obinutuzumab administered 2 weeks apart. Additional obinutuzumab infusions were administered during follow-up based on changes in clinical parameters |
| 5 | Cheng | 2025 | China | Retrospective cohort study | 40 | Immunologic remission defined as serum anti-PLA2R antibody titer < 2 RU/mL | Per 2021 KDIGO guidelines | Obinutuzumab initiated with intravenous infusion of 1 g. Following initial administration, repeated infusions of obinutuzumab were given at a single dose of 1 g. The interval between the first and second infusions was typically 2 weeks |
| 6 | Chen | 2025 | China | Single-arm clinical trail | 7 | Immunologic remission defined as serum anti-PLA2R antibody titer declining to < 14 RU/mL (ELISA) | Per 2021 KDIGO guidelines | Each obinutuzumab infusion was administered at a dose of 1000 mg, with at least a 2-week interval between doses. Infusion frequency was determined jointly by physician and patient following professional consultation |
| 7 | Sethi | 2020 | U.S. | Single-arm clinical trail | 10 | Not reported | Not reported | Not reported |
| 8 | Dong | 2025 | China | Single-arm clinical trail | 15 | Immunologic remission defined as seroconversion of PLA2R antibody (ELISA: PLA2R antibody titer < 14 RU/mL; indirect immunofluorescence: PLA2R antibody titer < 2 RU/mL) | Per 2021 KDIGO guidelines | Obinutuzumab was administered at a single dose of 1 g. The number of administrations was determined by clinicians based on KDIGO guideline recommendations and characteristics of individual patients. The interval between the first and second doses was typically 14 days; timing of the third dose was individualized according to B-cell levels |
| 9 | Su | 2024 | China | Single-arm clinical trail | 39 | Immunologic remission defined as serum anti-PLA2R antibody titer < 14 RU/mL (ELISA) | Per 2021 KDIGO guidelines | Patients received obinutuzumab infusions at a dose of 1000 mg per administration. The number of infusions was determined jointly by clinician and patient following professional discussion. The interval between the first and second infusions was typically 2–3 weeks, whereas the duration between the second and third infusions exceeded 6 months based on B-cell levels |
| 10 | Jha | 2025 | India | Single-arm clinical trail | 6 | Not reported | Not reported | Patients received 1 g intravenous obinutuzumab administered in two divided doses 14 days apart |
| 11 | Lin | 2024 | China | Single-arm clinical trail | 18 | Immunologic remission defined as serum anti-PLA2R antibody titer < 2 RU/mL | Per 2021 KDIGO guidelines | Patients received two 1000 mg doses of obinutuzumab administered 2 weeks apart. During follow-up, the initial cycle of obinutuzumab was concluded if CD19+ B-cell count reached BCD (defined as peripheral CD19+ B cells of 0 cells/μL). Otherwise, an additional 1 g dose was administered every 2 weeks until BCD was achieved |
| 12 | Sridharan | 2025 | Australia | Single-arm clinical trail | 5 | Immunologic remission defined as normalization of PLA2R titer | Complete remission defined as urinary PCR < 30 mg/mmol; partial remission defined as urinary PCR 30–300 mg/mmol with normalization of serum albumin | All patients received a standard obinutuzumab regimen comprising a 100 mg test dose on day 1, 900 mg on day 2, and 1000 mg on day 15 |

**Supplementary File 5** Rare adverse events

| Rare adverse events | No. of studies | No. of patients |
| --- | --- | --- |
| Palpitations | 3 | 4 |
| T-spot conversion | 2 | 1 |
| Dyspnea | 3 | 2 |
| Abnormality of hepatic function | 4 | 3 |
| The infection of soft tissues | 3 | 2 |
| Diaphoresis | 2 | 1 |
| Nausea and vomiting | 4 | 2 |
| Herpes zoster | 2 | 1 |
| Headache | 2 | 1 |
| Acute gastroenteritis | 2 | 1 |
| The infection of the biliary trac | 2 | 1 |
| Malaise | 2 | 1 |
| Amenorrhea | 2 | 1 |
| Tinnitus | 2 | 1 |
| Mortality | 2 | 1 |
